# Real-World Data on Access to Standards of Care for People With Spinal Muscular Atrophy in the UK

**DOI:** 10.3389/fneur.2022.866243

**Published:** 2022-05-30

**Authors:** Robert Muni-Lofra, Lindsay B. Murphy, Kate Adcock, Maria E. Farrugia, Joseph Irwin, James B. Lilleker, John McConville, Andria Merrison, Matt Parton, Liz Ryburn, Mariacristina Scoto, Chiara Marini-Bettolo, Anna Mayhew

**Affiliations:** ^1^John Walton Muscular Dystrophy Research Centre, Translational and Clinical Research Institute, Newcastle University and Newcastle Hospitals NHS Foundation Trust, Newcastle upon Tyne, United Kingdom; ^2^Department of Physical Therapy, Universitat Internacional de Catalunya, Barcelona, Spain; ^3^Muscular Dystrophy UK, London, United Kingdom; ^4^Institute of Neurological Sciences, Queen Elizabeth University Hospital, Glasgow, United Kingdom; ^5^TreatSMA, London, United Kingdom; ^6^Manchester Centre for Clinical Neuroscience, Northern Care Alliance NHS Foundation Trust, UK and Centre for Musculoskeletal Research, Manchester Academic Health Science Centre, The University of Manchester, Manchester, United Kingdom; ^7^Ulster Hospital, Belfast, United Kingdom; ^8^Bristol Neuromuscular Disease Centre, Bristol, United Kingdom; ^9^Queen Square Centre for Neuromuscular Diseases, University College London, London, United Kingdom; ^10^SMAUK, Stratford-upon-Avon, United Kingdom; ^11^Dubowitz Neuromuscular Centre, Great Ormond Street Hospital, London, United Kingdom

**Keywords:** spinal muscular atrophy, standards of care, neuromuscular diseases, real-world data, United Kingdom

## Abstract

Spinal Muscular Atrophy (SMA) is characterized by muscle atrophy and weakness and has an incidence of 1:11. 000 live births which projects an estimated population in the UK of 650–1,300 affected patients. Standards of Care (SoC) were updated in 2017 and they have been widely adopted as a reference for implementation of care in SMA across the globe. The effectiveness of implementation and adherence to these standards across different countries is unclear. The aim of this study is to describe the experience of individuals with SMA regarding their care in the UK. An online anonymised survey was sent out *via* patient organizations, the UK SMA Patient Registry, professional networks, and social media to reach across the UK. The survey captured demographic profile, professionals involved in a patient's care, Interventions and access to mobility aids and home adaptations. Participants responded about their access to services and to rate how important each professional and intervention was for their health and wellbeing. One hundred and twenty-eight responses were collected with a median age of 34 years (1–81). Seventy-three percent of participants were adults and 60% men. Overall good access to neurologist (>90%) but limited to nurse specialist (48%) and physiotherapist (57%). Good access to respiratory support was reported but limited for interventions for positioning and bracing and exercise. This survey highlights that access to certain professionals for people with SMA is limited in the UK. Striking differences were noted between pediatric and adult populations. Limited access to care were regularly reported, with half of the study population consistently not accessing full multidisciplinary care. Access to interventions for contracture management were recorded to have significant limitations. Mobility aids and home adaptations are widely available and were also reported as the most valued interventions. Access to nutritional support or speech and language therapy appears only to be available for a small proportion of the participants. Access to respiratory care was good especially in severe forms of SMA. We found pockets of good practice in the UK that align with the SoC. However, access is not equal for adults and children and access to certain professionals is significantly limited.

## Introduction

Spinal Muscular Atrophy (SMA) is characterized by muscle atrophy and weakness secondary to a degeneration of the motor neurons in the reduction of SMN protein (1). SMA has an incidence of 1:11.000 live births (2) and a prevalence of 1–2:100.000 (3) which projects an estimated population in the UK of 650–1,300 affected patients.

A Consensus document on Standards of Care (SoC) was published in 2007 (4) and updated in 2017 (5, 6). The aim of these publications was to benchmark diagnosis and management of SMA. The process was performed over different rounds of Delphi survey and was based on the available evidence for diagnosis and interventions (7–13) but also providing expert based recommendations and a consensus statement where new advances in care were not reflected in the existing literature (5).

Nine topics were included in the updated document: (1) Diagnosis and genetics; (2) Physical therapy and rehabilitation; (3) Orthopedic care, growth, and bone health care; (4) Nutrition; (5) Pulmonary care; (6) Acute care in the hospital setting; (7) Other organ system involvement; (8) Medication; (9) Ethics and palliative care. For all the relevant aspects of the condition a series of specific recommendations were made regarding management. These were presented as Neuromuscular and musculoskeletal evaluation, Rehabilitation, orthopedic management, Nutritional management, swallowing and gastrointestinal dysfunction and finally pulmonary management. All these topics were generally summarized with specific recommendations according to the different functional subtypes: non-sitters, sitters, and walkers (Table 1).

**Table 1:** Summary of recommendations on SoC.

**Neuromuscular and musculoskeletal evaluation**		**Assessment**
All		Assessments of **strength and range of joint motion, relevant motor functional scales and timed tests** to monitor those aspects of function that reflect activities of daily living. These assessments should be performed routinely by **trained examiners** every **6 months**.
**Rehabilitation**		
**Type**	**Assessment**	**Intervention**
Non-sitters	- Postural control - Scoliosis -Hip dislocation - Sitting tolerance - Chest deformities - Contractures ° ROM, goniometry - Muscle weakness ° antigravity movements - Functional Scales ° CHOP Intend - Motor development ° HINE	- *Positioning and bracing:* ° Daily use of seating systems, postural - *Stretching:* ° Daily use of orthosis (>60 min to overnight) ▪ Upper limb and AFO, KAFOS ° Braces (minimal frequency 5/week) ▪ TLSO ° Stretches (duration depending of specific patient needs) - *Promote function and mobility:* ° Seating and mobility systems ° Mobile arm supports for upper extremity function
Sitters	- Postural control - Foot and chest deformities - Scoliosis and pelvic obliquity - Hip dislocation - Contractures ° ROM, goniometry - Functional Scales ° HFMSE, RULM, MFM - Muscle weakness ° Strength tests	- *Positioning and bracing:* ° Thoracic bracing posture and promote function (minimal frequency 5 times/week) - Cervical bracing for safety and transportation - *Stretching:* - Daily use of orthosis (>60 min to overnight) - Stretches (Minimal frequency stretching 5–7/week) - Supported standing (up to 60 min, minimal frequency 3–5/week, optimal 5–7 times/week) - *Promote function and mobility:* ° Exercise for function, strength, ROM, endurance, ADLs, participation and balance ▪ Swimming, hippotherapy and wheelchair sport ° Electric/powered wheelchair with custom postural support ▪ Tilt/recline option and seta elevator sometimes necessary
Walkers	- Mobility - Timed tests - Measure of endurance ° 6 MWT - Falls - Functional Scales ° HFMSE, RULM - Muscle weakness ° Strength tests - Contractures ° ROM, goniometry - Postural control - Scoliosis - Hip dislocation	- *Positioning and bracing:* ° Lower limb orthosis for posture and function ° Thoracic bracing to promote posture in sitting - *Stretching:* ° Stretches (Minimal frequency stretching 2–3/week, optimal 3–5 times/week) - Use of orthoses according to specific needs °*Promote function and mobility:* ° Exercise (minimal frequency 2–3 times/week, optimal 3–5) ▪ Maintain flexibility and balance exercises
**Orthopedic Management**		
Non-sitters	- Cobb angle - Supine or sitting with trunk brace	° Spine deformity management ° Specific rigid braces
Sitters	- Inspection of spine - Spine radiographs - Hip instability - Contractures - Fractures	- Spinal orthoses (Rigid or soft orthoses) ° For scoliosis >20 degrees specially with significant growth remaining - Surgical intervention based on: ° Magnitude of curve (>50 degrees) ° Rate of progression (>10 degrees per year) ° Other factors ▪ Decreased respiratory function, parasol rib deformity, hyper kyphosis, pelvic obliquity, trunk imbalance) ° Delayed till age 4 years ° <8–10 years old: “growth-friendly” instrumentation ° 8–12 years old variability in practice
		- Hip instability: ° Only managed surgically in patients with significant pain - Contractures: ° Surgical management of contractures to be considered when caused pain or impair function - Fractures ° Closed treatment with cast for non-ambulant patients ▪ Avoid prolonger immobilization (> 4 weeks) ° Hip fractures: surgical stabilization
Walkers		- Fractures ° Long bone benefit from surgical stabilization
**Nutritional management, swallowing and gastrointestinal dysfunction**		
Non-sitters	- Optimal care: 3–5 months children, annually by adults - Video Fluoroscopic Swallow Study shortly after diagnosis - Difficulties feeding - Nutritional analysis of food records - Longitudinal anthropometrics	- Referral to specialist feeding therapy/modification - Nasojejunal tube until gastric-tube with Nissen fundoplication - Adjust caloric, fluid, macronutrient, micronutrient and timing of feeds - Minimize fasting during acute care (<6 h) - Monitor Fluid intake, electrolyte, glucose level. - Bowel regulation medications
Sitters	- Minimum: evaluation by dietician shortly after diagnosis - Optimal: evaluation every 3–6 months children, annually adults - Symptoms of dysphagia/aspiration/difficulties feeding - Video fluoroscopic swallow study if suggested by clinical signs - Nutritional analysis of food records - Longitudinal anthropometrics - Specific acute care monitoring	- If swallow safe, referral for feeding therapy/modifications - If swallow failed, nasofeeding tube- long term gastric feeding tube - Growth failure, supplemental nutrition products
Walkers	- Dietician for nutrition - Longitudinal anthropometrics	- Provide macro/micronutrient intakes based on guidelines for healthy sedentary individuals - Minimize fasting during acute care
**Pulmonary management**		
Non-sitters	- Initially every 3 months then 6 monthly - Hypoventilation (End tidal CO_2_) - Sleep study or pneumograms - Clinical assessment of gastroesophageal reflux	- Airway clearance with oronasal suction, physiotherapy/respiratory therapy, and cough augmentation to all non-sitters with ineffective cough - Ventilation for all symptomatic patients ° Some experts recommend it before documented respiratory failure ° Judge start based on clinical observation for adequate gas exchange or during sleep study ° NIV interfaces fitted by skilled physiotherapist - Customary immunizations, palivizumab and influenza + Mucolytics should not be used long-term
Sitters	−6 monthly - Same as above	- Same as above
Walkers	- Clinical evaluation for cough effectiveness or signs of hypoventilation	- Supportive care when needed - Customary immunizations, annual influenza and pneumococcal vaccination

*Adapted from Finkel et al. (14) and Mercuri et al. (5)*.

The SoC have been widely adopted as a reference for implementation of care in SMA across the globe. The guidelines have also been used as a benchmark for care during clinical trials (5) and more generally with these treatments more recently becoming available *via* clinical care. The paradigm shift in SMA treatments with the appearance of new disease modifying therapies (DMT) has raised some ethical questions on standardization of supportive care to evaluate its impact on DMT (14).

The implementation of these standards and adherence to them across different countries or regions is still unclear. Some studies have identified significant differences with implications on the age at which ambulation is lost (15). In the UK no information has been gathered as to the extent in which these SoC are being implemented or if care in the UK is meeting these standards. Evidence also suggests that there is a substantial psychosocial impact of living with SMA (16) which is an aspect of care that is not covered by the current SoC guidelines.

Understanding the extent to which SoC are implemented will help identifying potential gaps.

The aim of this study is to describe the experience of individuals living with SMA regarding their specialist care in the UK in relation to the SoC as described in published documents. This includes which health professionals they have access to, how often they are seen, access to interventions and management and patient satisfaction with their current level of care. In addition, information about psychological or emotional support and carers was added to capture aspects of care not described in the SoC documents.

## Methods

An online anonymized survey with a total of 31 questions was design on Survio (survio.com) for the purpose of this study. The link inviting individuals to participate in this survey was sent out *via* patient organizations, the UK SMA Patient Registry, professional networks, and social media to reach the SMA population across the UK. Given the nature of data collection—*via* voluntary participation in an online survey with no direct contact with the participants no consent was implied and therefore no ethical approval was required.

The survey was structured in four main topics:

- **Demographic** profile (*Questions 1–9*)

° Age, SMA type, functional status, and area of residence.

- **Range of professionals** involved in a patient's care (*Questions 10–13*)

° General Practitioner (GP), Pediatrician, Neurologist, Nurse Specialist, Physiotherapist, Occupational Therapist, Speech and Language Therapist, Pulmonologist, Respiratory Physiotherapist, Orthotist, Dietician/Nutritionist, Care Advisor, Carer and Psychologist/Counselor.

- **Interventions** that patients have access to (*Questions 14–26*)

° Contracture management (Splints, Stretches, etc.)
° Postural management (Braces, Standing devices, etc.)
° Respiratory support (NIV, cough augmentation)
° Exercise plan (Strengthening, Endurance, etc.)

- **Access to mobility aids and home adaptations** (*Questions 27–30*)

° Wheelchair access and home adaptations.

A final open text section was added for any additional comments.

Participants were asked about their access to services/care including location (*community, specialized center, or both*) and frequency of their visits. To gather their perception, participants were asked to rate how important each professional was for their health and wellbeing (*1 meaning not at all and 10, most important*). Participants were also asked to rate how often they would like to see each professional if applicable for them (*Less often, as much as I'm seen now, more often*).

To ascertain interventions and access to mobility aids and home adaptations a similar approach was performed. First participants were asked about their access to each specific intervention and if applicable, its frequency of use. Afterwards, the relevance was rated (1–10 as previously) and their degree of satisfaction about access was requested (*I don't need it, I believe I do need it but can't get it, I do need it and can get it but with limitations, I do need it and I get what I need*).

## Results

### Demographics

A total of 128 responses were collected (3 excluded due to a non-5q SMA diagnosis reported). The majority of participants (68%) took between 10 and 30 min to complete the survey. Overall completion rate was 21% (635 total visits) and none of the surveys were left incomplete.

Median age was 34 years (range from 1 to 81 years of age) with good representation across the different age ranges (average 9 responses per group) and with 73% of participants being adults and 60% men. Responses from participants below age 14 were collected through parents or tutors. Above that age, responses were reported by patient themselves or jointly with parents or carers.

**Figure 1:**
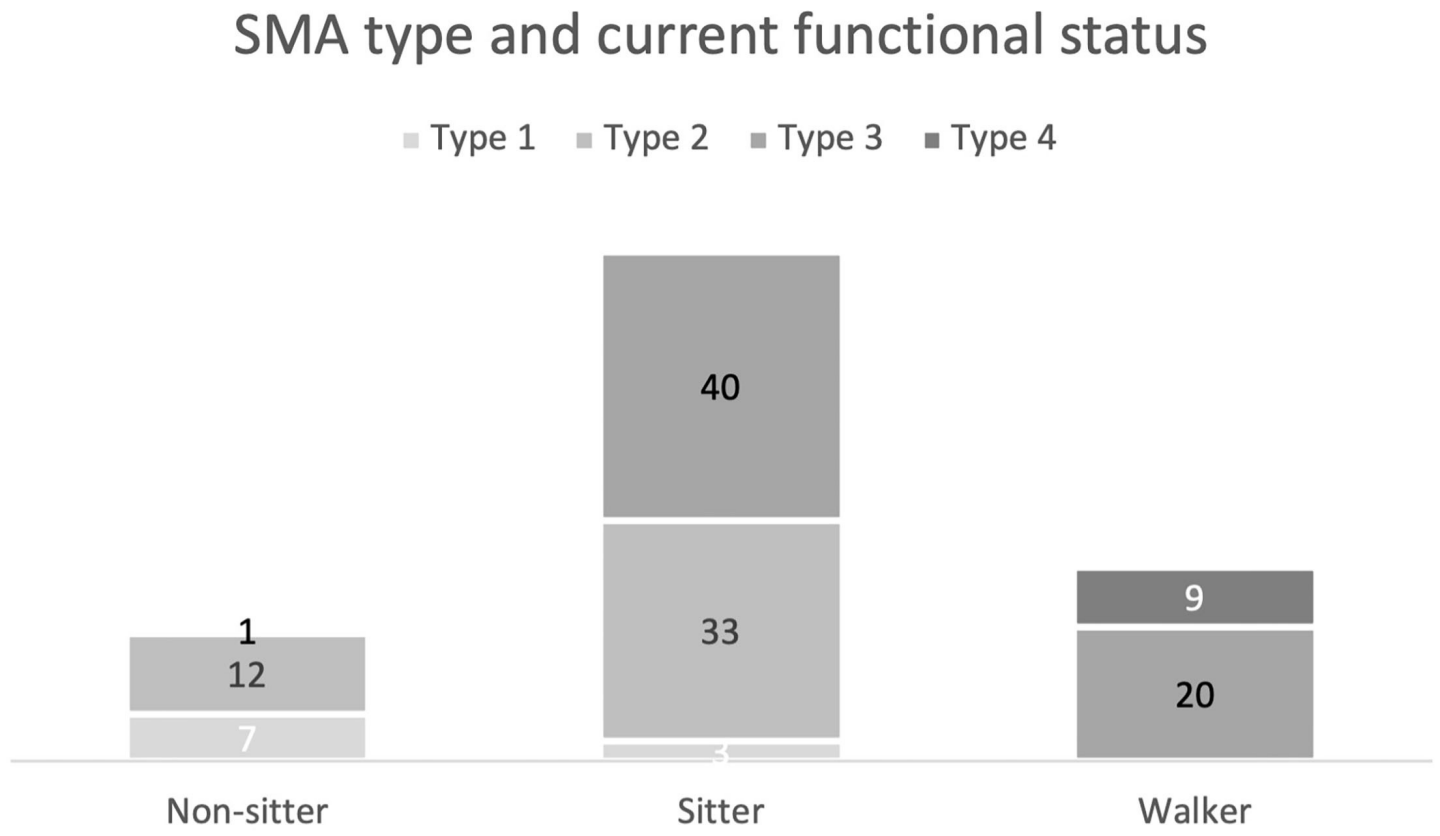
SMA type and current functional status distribution.

When analyzed by current functional status, sitters were the most represented functional group (76%) (Figure 1).

Most participants were based in England (85%) but also representation from Scotland, Wales, Northern Ireland, and Guernsey was collected. The sample from England was spread across 35 counties out of a total 48.

### Access to Professionals and Interventions According to Standards of Care

Access to SoC was measured by the proportion of participants that reported access to the relevant professional or intervention and how often they were seen or received the care.

*SoC recommendations include “neuromuscular and musculoskeletal evaluation by trained examiners every 6 months”*. (5) *Page 4*

A significant majority of the participants reported having access to a neurologist (Table 2A). This was consistent across age and functional ability except for walkers who reported having better access in the pediatric group. Sixty-four percent reported seeing a neurologist once or twice a year or more frequently however, a difference was observed between pediatric (94%) and adults (52%).

Table 2Reported access by age and functional group.

**Table:** 

**(A)**			
**Access reported to**	**Neurologist**	**Physiotherapist**	**Nurse**
**Pediatric**	**97%**	**100%**	**76%**
Non-sitter	100%	100%	89%
Sitter	95%	100%	84%
Walker	100%	100%	33%
**Adult**	**87%**	**41%**	**23%**
Non-sitter	91%	36%	45%
Sitter	93%	44%	26%
Walker	70%	45%	4%
**Grand total**	**90%**	**57%**	**48%**

**Table:** 

**(B)**				
**Access reported to**	**Splints**	**Spinal braces**	**Stretches**	**Supported standing**
**Pediatric**	**59%**	**15%**	**82%**	**53%**
Non-sitter	67%	22%	78%	33%
Sitter	53%	16%	89%	79%
Walker	67%	0%	67%	0%
**Adult**	**14%**	**13%**	**44%**	**7%**
Non-sitter	18%	18%	57%	0%
Sitter	14%	16%	47%	7%
Walker	13%	4%	30%	9%
**Grand total**	**26%**	**14%**	**54%**	**19%**

**Table:** 

**(C)**				
**Access reported to**	**Occupational therapist**	**Mobility aids**	**Home adaptations**	**Exercise**
**Pediatric**	**88%**	**91%**	**62%**	**91%**
Non-sitter	100%	89%	89%	78%
Sitter	89%	100%	47%	95%
Walker	63%	67%	67%	100%
**Adult**	**53%**	**86%**	**88%**	**62%**
Non-sitter	55%	100%	91%	73%
Sitter	63%	100%	98%	54%
Walker	26%	43%	61%	74%
**Grand total**	**62%**	**87%**	**81%**	**70%**

**Table:** 

**(D)**		
**Access reported to**	**Dietician/nutritionist**	**Speech and language therapist**
**Pediatric**	**47%**	**44%**
Non-sitter	67%	78%
Sitter	42%	36%
Walker	33%	0%
**Adult**	**11%**	**10%**
Non-sitter	36%	27%
Sitter	11%	10%
Walker	0%	0%
**Grand total**	**21%**	**16%**

**Table:** 

**(E)**				
**Access reported to**	**Pulmonologist**	**Respiratory physiotherapist**	**Ventilator**	**Airway clearance**
**Pediatric**	**60%**	**54%**	**32%**	**38%**
Non-sitter	100%	100%	89%	89%
Sitter	53%	53%	11%	26%
Walker	29%	0%	17%	0%
**Adult**	**42%**	**26%**	**19%**	**21%**
Non-sitter	82%	73%	73%	64%
Sitter	42%	27%	16%	21%
Walker	22%	0%	0%	0%
**Grand total**	**47%**	**34%**	**22%**	**26%**

*The bold values indicates the overall figures for pediatric, adult and grand total of the cohort (in opposition to breakdown by functional status for the same groups)*.

Access to a nurse specialist was reported by less than half of the participants again showing a discrepancy between pediatric (76%) and adult (23%) responders. Frequency of visits was reported to be once or twice a year or more by 57%.

In total, over half of the participants reported having access to a physiotherapist with a significant difference over pediatric and adult responders. Sixty-four percent of participants reported seeing a physiotherapist once a year or more (81% pediatric, 38% adult). Only 14% reported regular access (once every 2 months or more) with a difference for age group (38% pediatric, 5% adult).

*In the Rehabilitation section, recommendations are made for “positioning and bracing”. These include: use of orthosis (splints) for more than 60 min or overnight, use of braces for non-sitters and sitters 5 times a week*. (5) *Page 5*

The use of splints was reported by just over quarter of all participants with non-sitters using them most compared to sitters and walkers (Table 2B). The frequency and duration of use was reported to be for an hour a day or more by 43% of the users—non-sitters (63%) followed by walkers (55%) and sitters (30%).

Spinal braces were reported to be used by a minority of the overall participants being mainly non-sitters and sitters with 73% reporting using them more than 3 h a day.

*Also in the rehabilitation section, stretches are recommended with different regimes depending on functional status: to be adapted to patients needs for non-sitters, 5–7 times a week for sitters and 2–3 to 3–5 times a week for walkers*. (5) *Page 5*

Over half of the participants reported doing stretches with higher rate for non-sitters, followed by sitters and walkers (Table 2B). Looking at performing stretches by age group, pediatric participants have a higher rate than adults.

*The use of supported standing devices is recommended in addition to stretches for sitters for 60 min, 3–5 to 5–7 times a week*. (5) *Page 5*

A supported standing device (Standing frame or KAFOS) is used by a minority of participants (Table 2B); 20% of these reported using this device for an hour a day or more as recommended in SoC. The most commonly reported use was for an hour almost every day (43% of the users).

*In the rehabilitation section several interventions are recommended to “promote function and mobility”. Introduction of home adaptations, mobility aids and exercises are recommended with different specification depending on functional type. It is suggested that exercise can have an effect on function, strength, ROM, endurance for sitters. Walkers are encouraged to perform aerobic and general conditioning exercise (at least for 30 min per session). Some examples of specific exercises are suggested for both types including swimming hippotherapy, wheelchair sports, walking, rowing, cycling, yoga, etc*. (5) *Page 5*

Access to occupational therapy is reported to be available to over half of the participants with much higher proportion in pediatrics in comparison to adults (Table 2C). Frequency of once a year or more was reported by 26% of the users with “being seen when needed” the most common response (65% overall, 50% of pediatric, 73% of adults).

Mobility aids and home adaptations are accessible to the great majority of the participants with higher access for more severe phenotypes (Table 2C).

Home adaptations are spread across different dimensions. Fifty-four percent of participants have access to mobility adaptations (handrails, stair lifts, ramps, etc), 62% for selfcare (toilet, shower, wet room, etc), 48% for transfers (hoist, sliding board, etc.) (75% for non-sitters, 57% of sitters and 7% walkers) and 22% accessories (adapted cutlery, trays, adapted clothes, etc.) (30% of non-sitters, 26% of sitters and 7% walkers).

Access to any form of exercise was reported by most of the participants with much higher rate for pediatrics (Table 2C). Endurance exercise was accessible for 20% (5% of non-sitters, 17% of sitters, 38% of walkers and 38% of pediatrics, 13% of adults). Mixed exercise (yoga, Pilates, etc.) was accessible by 6% of the participants (9% pediatric and none of the adults).

*In the Nutrition section, optimal evaluation was recommended to be for non-sitters and sitters from 3 to 6 months for children and yearly for adults*. (5) *Page 8*

Access to dietician or nutritionist and speech and language therapist is reported to be available to a minority of the participants with more than double proportion for pediatric in comparison to adults. Access was more present also for more severe phenotypes (Table 2D). The frequency most reported for visits to dietician or nutritionist was “when needed” for pediatrics (69%) and once or twice a year for adult users (60%).

For speech and language therapist, 35% of the users reported to be seen at least once a year in (38% pediatrics, 30% adults), with “being seen when needed” the most common result for pediatrics (62%) and less than once a year (40%) for adults.

*In the respiratory section, SoC recommendation suggest regular assessment for non-sitters (3–6 monthly) and sitters (6 monthly) and access when needed for walkers. It is also recommended access to support for airway clearance, physiotherapy/respiratory therapy and ventilation for all symptomatic patients*. (6) *Page 3*

Access to pulmonologist is reported by nearly half of the participants and in slightly lower proportion for respiratory physiotherapist (Table 2E). There were differences for both professionals when comparing pediatric and adult participants. As seen previously, access was also higher for more severe phenotypes being nearly unexciting for walkers. Frequency of visits was reported to be for once or twice a year or higher in 80% of the users for pulmonologist and by 59% for respiratory physiotherapist.

Access to ventilation and airway clearance is reported by nearly a quarter of the participants with differences by functional level (Table 2E). Again, more severe phenotype reported higher rate of access. For ventilation, the most common frequency if use was “every night” (48%) with 33% of non-sitters reporting additional daytime use. For airway clearance the frequency of use most reported was “twice a day” by non-sitters (47%) and “when needed” by sitters (41%).

In addition to the professionals included in the SoC document, access to psychological support was reported to as available by 14% of the participants with a reported frequency of visits “when needed” by 44%.

### Participant's Perception

Participants rated the importance of having access to different professionals and interventions represented by age and functional group (Tables 3, 4).

**Table 3:** Rate of importance by professionals by age and functional group.

**Mean, SD**	**General** **practitioner**		**Pediatrician**		**Neurologist**		**Nurse** **specialist**		**Physiotherapist**		**Occupational** **therapist**		**Speech and** **language** **therapist**		**Pulmonologist**		**Respiratory** **physio** **-therapist**		**Orthotist**		**Dietician/** **nutritionist**		**Care** **advisor**		**Carer**		**Psychologist/** **emotional** **support/** **counselor**	
**Pediatric**	**5.1**	**2.9**	**5.4**	**3.4**	**8.9**	**2.2**	**6.7**	**3.2**	**9.2**	**1.6**	**8.6**	**2.0**	**3.4**	**3.5**	**5.8**	**4.0**	**5.9**	**4.1**	**7.7**	**2.5**	**4.7**	**3.5**	**4.1**	**4.0**	**2.6**	**3.3**	**3.1**	3.2
Non-sitter	5.3	2.6	5.3	3.5	8.6	2.6	7.4	2.7	8.6	1.9	7.7	2.8	4.9	3.8	8.3	2.2	8.4	2.2	6.4	2.4	4.7	3.2	3.4	3.9	5.0	4.7	2.2	2.2
Sitter	5.5	3.2	5.9	3.6	8.9	2.3	7.6	2.7	9.9	0.3	9.0	1.7	3.5	3.6	5.7	4.3	6.2	4.2	8.0	2.8	4.4	3.6	4.3	4.1	1.8	2.3	2.4	2.6
Walker	3.8	2.2	3.8	2.4	9.0	1.7	2.8	2.9	7.8	2.4	8.5	0.8	1.0	0.0	2.3	2.2	1.2	0.4	8.5	1.0	6.0	4.1	4.7	4.4	1.3	0.8	6.8	3.7
**Adult**	**6.4**	**2.9**	**1.8**	**2.3**	**7.9**	**2.4**	**3.9**	**3.4**	**6.7**	**3.4**	**5.6**	**3.2**	**2.4**	**2.5**	**5.2**	**3.9**	**4.3**	**3.8**	**3.0**	**3.0**	**4.0**	**3.5**	**3.8**	**3.5**	**6.2**	**4.3**	**4.4**	3.6
Non-sitter	7.8	1.7	2.5	3.4	8.2	1.9	4.5	3.5	6.0	3.4	5.5	2.8	2.8	2.8	8.9	1.6	6.9	3.3	4.2	3.8	5.3	3.6	3.6	3.3	9.1	2.7	5.0	3.8
Sitter	6.6	2.9	1.9	2.4	7.9	2.4	4.7	3.6	7.1	3.4	6.2	3.0	2.7	2.8	5.7	3.9	5.0	4.0	2.9	2.9	4.5	3.6	4.5	3.6	6.9	4.1	4.7	3.7
Walker	5.3	3.1	1.3	1.5	7.9	2.6	1.8	1.9	6.2	3.4	4.4	3.6	1.3	0.9	2.1	2.3	1.4	1.0	2.4	2.8	2.4	2.9	2.0	2.7	2.9	3.4	3.4	3.5
**Total**	**6.1**	**2.9**	**2.8**	**3.1**	**8.2**	**2.4**	**4.7**	**3.6**	**7.4**	**3.2**	**6.4**	**3.2**	**2.6**	**2.8**	**5.4**	**3.9**	**4.8**	**3.9**	**4.2**	**3.6**	**4.2**	**3.5**	**3.9**	**3.6**	**5.2**	**4.3**	**4.1**	3.6

*1 (black) = not at all important and 10 (white) = most important. The bold values indicates the overall figures for pediatric, adult and grand total of the cohort (in opposition to breakdown by functional status for the same groups)*.

**Table 4:** Rate of importance by intervention by age and functional group.

**Mean, SD**	**Splints**		**Back braces**		**Supported standing**		**Ventilator**		**Cough augmentation**		**Stretches**		**Streng** **-thening exercises**		**Endurance exercise**		**Mixed exercise**		**Other exercise**		**Mobility devices**		**Home adaptations**	
**Pediatric**	**7.2**	**3.2**	**4.3**	**4.2**	**6.3**	**4.0**	**4.4**	**4.1**	**5.2**	**4.4**	**9.0**	**1.2**	**8.6**	**2.5**	**7.8**	**3.0**	**6.8**	**3.6**	**6.4**	**3.9**	**9.5**	**1.8**	**9.1**	**2.0**
Non-sitter	7.0	2.7	3.6	4.4	6.0	3.7	8.9	1.7	9.5	1.1	8.7	1.1	8.0	3.2	7.6	3.3	7.9	3.2	5.6	4.3	9.0	3.0	9.4	1.3
Sitter	7.5	3.2	5.5	4.3	7.5	3.6	2.4	3.1	4.2	4.3	9.4	1.1	8.8	2.5	7.8	3.1	7.2	3.5	7.5	3.6	9.9	0.2	9.4	2.1
Walker	6.2	4.1	1.0	0.0	1.0	0.0	2.8	3.5	1.0	0.0	7.8	1.3	8.4	1.5	8.2	2.7	4.0	3.9	3.8	4.1	8.8	2.0	7.8	2.4
**Adult**	**2.9**	**3.1**	**2.3**	**3.1**	**2.4**	**2.9**	**3.4**	**3.7**	**4.0**	**4.1**	**6.8**	**3.2**	**6.7**	**3.3**	**5.9**	**3.7**	**5.3**	**3.8**	**5.6**	**3.9**	**8.4**	**3.2**	**9.0**	**2.1**
Non-sitter	4.2	4.1	2.8	3.6	2.3	3.0	8.1	3.2	7.9	3.4	7.4	3.0	5.8	3.8	4.7	3.9	3.8	3.2	4.4	3.6	10	0.0	8.9	2.0
Sitter	2.8	2.9	2.8	3.5	2.8	3.2	3.2	3.6	4.3	4.2	7.0	3.2	6.6	3.4	5.4	3.9	5.4	4.0	5.9	4.0	9.7	1.4	9.7	1.0
Walker	2.6	3.2	1.0	0.0	1.5	2.1	1.0	0.0	1.0	0.0	6.1	3.3	7.4	2.9	7.5	2.7	6.0	3.7	5.7	3.9	4.5	3.8	7.3	3.2
**Total**	**4.2**	**3.7**	**2.9**	**3.5**	**3.6**	**3.7**	**3.7**	**3.8**	**4.3**	**4.2**	**7.5**	**2.9**	**7.3**	**3.2**	**6.5**	**3.6**	**5.8**	**3.8**	**5.9**	**3.9**	**8.7**	**2.9**	**9.0**	**2.1**

*1 (black) = not at all important and 10 (white) = most important. The bold values indicates the overall figures for pediatric, adult and grand total of the cohort (in opposition to breakdown by functional status for the same groups)*.

Participant's perception about current access was also captured with scores ranging from not applicable, satisfied with current access or access with limitations. For the professional this was reported with the options “would like to see them” more often or less often. The option “less often” was only reported by one individual consistently across different professionals involved. This option has been excluded from the table to limit the presence of a column with minimal significance.

Over half of the participants reported satisfactory access to a neurologist with only a minority reporting they role wasn't applicable to them (Table 5A). The was a difference when comparing pediatric participants to adults.

**Table 5:** Reported frequency of access satisfaction by age and functional group.

**(A)**																
	**Neurologist**			**Nurse specialist**			**Physiotherapist**			**Occupational therapist**						
**Frequency satisfaction**	**More often**	**Not applicable for me**	**As much as I'm seen now**	**More often**	**Not applicable for me**	**As much as I'm seen now**	**More often**	**Not applicable for me**	**As much as I'm seen now**	**More often**	**Not applicable for me**	**As much as I'm seen now**				
**Pediatric**	**26%**	**9%**	**66%**	**23%**	**23%**	**54%**	**57%**	**3%**	**40%**	**23%**	**9%**	**69%**				
Non-sitter	11%	11%	78%	11%	11%	78%	67%	0%	33%	22%	0%	78%				
Sitter	26%	11%	63%	21%	21%	58%	53%	5%	42%	21%	11%	68%				
Walker	43%	0%	57%	43%	43%	14%	57%	0%	43%	29%	14%	57%				
**Adult**	**49%**	**5%**	**46%**	**23%**	**60%**	**17%**	**66%**	**16%**	**17%**	**34%**	**32%**	**35%**				
Non-sitter	36%	0%	64%	18%	55%	27%	36%	18%	45%	45%	18%	36%				
Sitter	47%	5%	48%	29%	51%	20%	76%	12%	12%	36%	22%	41%				
Walker	61%	9%	30%	9%	87%	4%	57%	26%	17%	22%	61%	17%				
**Grand total**	**43%**	**6%**	**51%**	**23%**	**50%**	**27%**	**64%**	**13%**	**24%**	**31%**	**25%**	**44%**				
**(B)**																
	**Dietician/nutritionist**			**Speech and language therapist**			**Pulmonologist**			**Respiratory physiotherapist**						
**Frequency satisfaction**	**More often**	**Not applicable for me**	**As much as I'm seen now**	**More often**	**Not applicable for me**	**As much as I'm seen now**	**More often**	**Not applicable for me**	**As much as I'm seen now**	**More often**	**Not applicable for me**	**As much as I'm seen now**				
**Pediatric**	**20%**	**46%**	**34%**	**3%**	**69%**	**29%**	**6%**	**43%**	**51%**	**20%**	**43%**	**37%**				
Non-sitter	22%	33%	44%	11%	33%	56%	0%	0%	100%	33%	0%	67%				
Sitter	16%	53%	32%	0%	74%	26%	11%	47%	42%	21%	42%	37%				
Walker	29%	43%	29%	0%	100%	0%	0%	86%	14%	0%	100%	0%				
**Adult**	**29%**	**60%**	**11%**	**8%**	**82%**	**11%**	**20%**	**49%**	**32%**	**23%**	**57%**	**21%**				
Non-sitter	30%	20%	50%	0%	64%	36%	27%	0%	73%	27%	18%	55%				
Sitter	36%	56%	8%	9%	81%	10%	24%	44%	32%	27%	51%	22%				
Walker	13%	87%	0%	9%	91%	0%	5%	86%	9%	9%	91%	0%				
**Grand total**	**27%**	**56%**	**17%**	**6%**	**78%**	**16%**	**16%**	**47%**	**37%**	**22%**	**53%**	**25%**				
**(C)**																
	**Splints**				**Spinal braces**				**Stretches**				**Supported standing**			
**Frequency satisfaction**	**I believe I do need it but can't get it**	**I do need it and can get it but with limitations**	**I do need it and I get what I need**	**I don't need it**	**I believe I do need it but can't get it**	**I do need it and can get it but with limitations**	**I do need it and I get what I need**	**I don't need it**	**I believe I do need it but can't get it**	**I do need it and can get it but with limitations**	**I do need it and I get what I need**	**I don't need it**	**I believe I do need it but can't get it**	**I do need it and can get it but with limitations**	**I do need it and I get what I need**	**I don't need it**
**Pediatric**	**12%**	**21%**	**38%**	**29%**	**3%**	**15%**	**12%**	**71%**	**9%**	**41%**	**44%**	**6%**	**6%**	**12%**	**38%**	**44%**
Non-sitter	0%	33%	33%	33%	0%	22%	0%	78%	11%	33%	33%	22%	**11%**	**22%**	**11%**	**56%**
Sitter	16%	16%	42%	26%	5%	16%	21%	58%	5%	42%	53%	0%	**5%**	**11%**	**63%**	**21%**
Walker	17%	17%	33%	33%	0%	0%	0%	100%	17%	50%	33%	0%	**0%**	**0%**	**0%**	**100%**
**Adult**	**8%**	**12%**	**8%**	**73%**	**3%**	**5%**	**5%**	**86%**	**26%**	**29%**	**19%**	**26%**	**10%**	**3%**	**5%**	**81%**
Non-sitter	18%	0%	18%	64%	9%	0%	9%	82%	27%	27%	18%	27%	**9%**	**0%**	**0%**	**91%**
Sitter	9%	12%	7%	72%	4%	9%	7%	81%	28%	32%	18%	23%	**14%**	**5%**	**5%**	**75%**
Walker	0%	17%	4%	78%	0%	0%	0%	100%	22%	22%	22%	35%	**0%**	**0%**	**9%**	**91%**
**Grand total**	**9%**	**14%**	**16%**	**61%**	**3%**	**8%**	**7%**	**82%**	**22%**	**32%**	**26%**	**21%**	**9%**	**6%**	**14%**	**71%**
**(D)**																
	**Mobility aids**				**Home adaptations**				**Ventilation**				**Cough augmentation**			
**Frequency satisfaction**	**I believe I do need it but can't get it**	**I do need it and can get it but with limitations**	**I do need it and I get what I need**	**I don't need it**	**I believe I do need it but can't get it**	**I do need it and can get it but with limitations**	**I do need it and I get what I need**	**I don't need it**	**I believe I do need it but can't get it**	**I do need it and can get it but with limitations**	**I do need it and I get what I need**	**I don't need it**	**I believe I do need it but can't get it**	**I do need it and can get it but with limitations**	**I do need it and I get what I need**	**I don't need it**
**Pediatric**	**6%**	**44%**	**44%**	**6%**	**29%**	**26%**	**38%**	**6%**	**0%**	**6%**	**35%**	**59%**	**6%**	**9%**	**29%**	**56%**
Non-sitter	0%	56%	33%	11%	22%	33%	44%	0%	0%	11%	89%	0%	11%	11%	67%	11%
Sitter	0%	42%	53%	5%	26%	26%	37%	11%	0%	5%	16%	79%	5%	11%	21%	63%
Walker	33%	33%	33%	0%	50%	17%	33%	0%	0%	0%	17%	83%	0%	0%	0%	100%
**Adult**	**1%**	**48%**	**37%**	**13%**	**16%**	**46%**	**30%**	**8%**	**0%**	**0%**	**19%**	**81%**	**8%**	**1%**	**23%**	**68%**
Non-sitter	0%	64%	36%	0%	18%	55%	27%	0%	0%	0%	64%	36%	9%	0%	73%	18%
Sitter	0%	58%	39%	4%	16%	53%	30%	2%	0%	0%	18%	82%	11%	2%	23%	65%
Walker	4%	17%	35%	43%	17%	26%	30%	26%	0%	0%	0%	100%	0%	0%	0%	100%
**Grand Total**	**2%**	**47%**	**39%**	**11%**	**20%**	**41%**	**32%**	**7%**	**0%**	**2%**	**23%**	**75%**	**7%**	**3%**	**25%**	**65%**
**(E)**																
	**Strengthening exercises**				**Endurance exercises**				**Mixed exercises (Ioga, Pilates)**							
**Frequency satisfaction**	**I believe I do need it but can't get it**	**I do need it and can get it but with limitations**	**I do need it and I get what I need**	**I don't need it**	**I believe I do need it but can't get it**	**I do need it and can get it but with limitations**	**I do need it and I get what I need**	**I don't need it**	**I believe I do need it but can't get it**	**I do need it and can get it but with limitations**	**I do need it and I get what I need**	**I don't need it**				
**Pediatric**	**15%**	**44%**	**32%**	**9%**	**21%**	**35%**	**26%**	**18%**	**21%**	**15%**	**6%**	**59%**				
Non-sitter	11%	33%	22%	33%	22%	22%	22%	33%	33%	0%	0%	67%				
Sitter	11%	53%	37%	0%	16%	42%	26%	16%	11%	16%	11%	63%				
Walker	33%	33%	33%	0%	33%	33%	33%	0%	33%	33%	0%	33%				
**Adult**	**36%**	**25%**	**15%**	**23%**	**34%**	**19%**	**15%**	**32%**	**24%**	**13%**	**10%**	**53%**				
Non-sitter	27%	27%	0%	45%	27%	18%	0%	55%	9%	0%	9%	82%				
Sitter	40%	26%	12%	21%	40%	16%	9%	35%	26%	16%	7%	51%				
Walker	30%	22%	30%	17%	22%	26%	39%	13%	26%	13%	17%	43%				
**Grand total**	**30%**	**30%**	**20%**	**19%**	**30%**	**23%**	**18%**	**28%**	**23%**	**14%**	**9%**	**54%**				

*The bold values indicates the overall figures for pediatric, adult and grand total of the cohort (in opposition to breakdown by functional status for the same groups)*.

Access to nurse specialist was reported to be not applicable by half of the participants being much higher for adult participants when compared to pediatrics. This differences made that most of the pediatric participants were satisfied with current access and most adults reported the role not applicable for them. Most participants reported insufficient access to physiotherapist with slightly higher rate within adult participants. Access to occupational therapist was nearly splint in thirds for each category being satisfactory access the most reported one. This proportion was higher for pediatric participants.

Most of participants rated access to a dietician/nutritionist and speech and language therapist as not applicable with small differences in between pediatric patients and adults (Table 5B).

Over half reported that access to a Pulmonologist was applicable with slightly higher proportion of adult willing to see them more often (Table 5B). By functional status, the role had clear trends for non-sitters where they had satisfactory access and walkers that find the role not relevant. When looking at sitters, there more spread across the three categories with predominance of the role not being applicable for nearly half of the participants. Respiratory Physiotherapist access follows a similar pattern with slightly higher rate of unsatisfied participants (Table 5B).

Forty-eight percent participants reported that access to psychologist or emotional support was applicable, with 38% willing to see them more often. Of those accessing this support 79% said that would like to receive this support more frequently either for themselves or their child.

Participant's perception around access to specific interventions is reported again with the most common option selected with additional distinction by age group or functional status when significant differences were noted.

Most of the participants reported not needing access to splints with a significant contribution of adult participants (Table 5C). When looking at the proportion separately, the majority of pediatric patient reported to get what they need.

Access to spinal braces was perceived as not needed by most of the participants being only pediatric sitters the ones to report higher rates for satisfactory access and access with limitations.

Access to stretches was perceived as needed by the majority reporting a similar degree of satisfaction with current access across different functional status. Pediatric patients' higher degree of satisfaction age group.

Most of the participants reported not needing access to a supported standing devices, with much higher proportion for adults. This was particularly true for walkers being sitters the functional group with higher degree of satisfaction.

Access to mobility devices was reported to be widely accessible with a similar distribution for those who have access with some limitations and those who have access to what they need (Table 5D). There were no major differences in between functional or age group with the only exception of adults walkers where the majority reported not needing mobility aids.

For home adaptations the distribution of responses was similar to mobility aids but showing higher rate of participants with no access.

Access to Ventilation was reported as accessible when needed with a small proportion having access with limitations. It was clearly less needed for less severely affected participants. Airway clearance devices follow a similar pattern but with a higher rate of participants reporting no access despite needing it (Table 5E).

In relation to access to exercise, endurance, strengthening and mixed exercise were reported as not being accessible by a similar proportion of participants with slightly higher rates for adults (Table 3E). Access with limitations or satisfactory access was reported to be higher in pediatric patients for strengthening and endurance exercise, whilst having similar figures for mixed exercise. The proportion of participants that reported not needing each form of exercise, it was again higher for adults and gradually increase overall from strengthening, endurance to mixed having the highest proportion.

## Discussion

This study made use of an online survey technique to capture participants who were representative of different areas, ages and SMA types. The overall response rate of 21% which is slightly lower than reference values of 25–30% (17) was considered acceptable in the context of rare diseases. The sample included individuals aged between 1 and 81 years with a bias toward adult participants over pediatric ones. In relation to the SMA type, type 3 seems to be overrepresented when compared to the current figures from different registries where type 2 is often the more represented type (15). One of the potential explanations of this bias is since during the time that the survey was open (August 2020–April 2021), managed access agreement didn't include SMA type 3. This was perceived from patient organization as one of the potential explanations for the higher participation rate in the survey.

The SoC for SMA defines which professionals should be accessible to individuals with this condition. Our survey highlights that, certain professionals are not accessible to patients and underscores the striking differences in access to certain specialties between pediatric and adult patient populations. Figures range from 59% difference for access to physiotherapy (100% pediatric, 41% adults) to 15% difference for access to a neurologist (85% pediatric and 70% adults). This holds true for access to interventions, ranging from 46% difference for access to supported standing (53% pediatric, 7% adult) and 45% difference for access to splints (59% pediatric, 14% adult) to 5% difference for access to mobility aids (91% pediatric, 86% adults). Because SMA is a progressive disease regardless of age (18), this implies that these differences will ultimately create a significant gap in care and provision for adults with SMA. However, this is not to say that access meets the SoC in children although the level of care is better. On the other hand, access to specific professional or interventions follow a clear pattern that correlates with disease severity. Access to pulmonologist and respiratory physiotherapist are a good example of this.

Limited access to care and provision recommended within the SoC document were regularly reported, with half of the study population consistently not accessing full multidisciplinary care. Regular follow up by a neurologist was accessible by most of the participants but more limited to other members of the MDT team (nurse specialist and physiotherapist). When looking at the frequency of visits, only around 65% of the participants are seen once or twice a year which confirms, even for those accessing specialists such as neurologists that SoC are unfortunately not being met.

The SoC document outlines the importance of access to interventions for contracture management however, this study highlights significant limitations to this access. This is particularly evident around access to spinal braces and supported standing which was only available to <20% of the participants but also for splints (26%). It would appear from this data that if a patient has access to a spinal brace or standing device that they are likely to make use of them. However, this is less true if you are provided with splints. This poor uptake of use may be associated with limited capacity for follow up from multidisciplinary team as highlighted above (i.e., follow up to ensure good fit).

Performing stretches is probably one of the clearer examples of an intervention where it is difficult to predict the specific needs for specific age groups or even specific individuals, or patients with a particular functional status. However, only 17% of the participants reported doing more than 3 h a week of stretches. There are different reasons that might influence the limited undertaking of these interventions but is also important to identify factors that might limit the relevant support required to ensure its recommended use such as access to more regular physiotherapy.

Exercise is widely accessible for many survey participants, but limited frequency of use raises questions as to why those with SMA do not exercise more frequently. In a similar way that the performance of stretches can be limited due to limited support, access to adapted facilities within a relatively short distance of patients can be a significant factor to limits other forms of exercise. Exercise, in its many different forms, was highly valued by participants which infers an understanding of the benefit of exercise among the SMA population and therefore may have great potential for improvement in this aspect of care.

Mobility aids and home adaptations appeared to be widely available and were also reported as the most valued type of intervention across age and functional groups. Access to occupational therapy was reported as being limited but 65% reported they had access when needed which might be the explanation for the good accessibility to mobility aids and the relevant home adaptations as in the UK occupational therapists are often providers of mobility and adaptations rather than providing specific support and practice for activities of daily living.

Access to nutritional support or speech and language therapy appears only to be available for a small proportion of the participants. The fact that this access decreases with age and disease severity is of some concern given the importance of these interventions within the SoC document.

Access to respiratory care was good especially when looking at the more severe forms of SMA, which is reassuring due to the predominance of respiratory issues as the disease progresses (19). However, limited access or no access to cough augmentation was reported by 15% of the non-sitters and sitters which raises the question of equitable access across the UK. Due to the limited representation of participants from each region of the UK it is not possible to identify if this proportion of participants is representative of specific regions of the country.

One of the main limitations of this study is the small sample recruited in comparison to the estimated SMA population in the UK. Up to 3 attempts were undertaken to reach the targeted population through patient registry, patient organizations and social media and increase participation. The limited number of responders may skew the results as the methods used will not include those with no access to technology. It is difficult therefore to infer this survey population is truly representative of the overall population of SMA in the UK, however clear trends within age groups and functional status were observed.

This study also suggests the need of further studies to gain a better understanding of the limiting factors for contracture management. It is important to identify potential solution related to training needs, additional budget allocated to community services or the increase overall awareness about SMA. This is crucial due to the impact of these aspects of care in conjunction with disease modifying treatments. For similar reasons it is recommended to undertake further investigations around effects and uptake of exercise people with individuals with SMA.

There are pockets of good practice in the UK such as access to respiratory care or neurologist that align with the standards of care documents. However, access is not equal for adults and children and access to certain healthcare professionals like physiotherapist, SALT or nutritionist is significantly limited. This creates a limitation in supportive care which is not reflected by the natural history of the disease.

Exercise and rehabilitation are particularly important to maximize the benefits of disease modifying therapies. This is particularly relevant not only to have access but to have the supportive care to ensure consistency in their practice. From this study it is clear that this is not in place for the UK.

## Data Availability Statement

The raw data supporting the conclusions of this article will be made available by the authors, without undue reservation.

## Ethics Statement

Ethical review and approval was not required for the study on human participants in accordance with the local legislation and institutional requirements. Written informed consent from the participants' legal guardian/next of kin was not required to participate in this study in accordance with the national legislation and the institutional requirements.

## Author Contributions

RM-L, CM-B, and AMa contributed to the concept and design of the study. RM-L and AMa wrote the first draft of the manuscript. All authors contributed to manuscript revision, read, and approved the submitted version.

## Conflict of Interest

LR was employed by SMAUK. JI was employed by Treat SMA and KA was employed by MDUK. The remaining authors declare that the research was conducted in the absence of any commercial or financial relationships that could be construed as a potential conflict of interest.

## Publisher's Note

All claims expressed in this article are solely those of the authors and do not necessarily represent those of their affiliated organizations, or those of the publisher, the editors and the reviewers. Any product that may be evaluated in this article, or claim that may be made by its manufacturer, is not guaranteed or endorsed by the publisher.
